# Bile acid receptors and renal regulation of water homeostasis

**DOI:** 10.3389/fphys.2023.1322288

**Published:** 2023-11-15

**Authors:** Yanlin Guo, Taotao Luo, Guixiang Xie, Xiaoyan Zhang

**Affiliations:** ^1^ Division of Nephrology, Wuhu Hospital, East China Normal University, Wuhu, China; ^2^ Health Science Center, East China Normal University, Shanghai, China

**Keywords:** FXR, tgr5, aquaporin, kidney, water homeostasis

## Abstract

The kidney is the key organ responsible for maintaining the body’s water and electrolyte homeostasis. About 99% of the primary urine filtered from the Bowman’s capsule is reabsorbed along various renal tubules every day, with only 1–2 L of urine excreted. Aquaporins (AQPs) play a vital role in water reabsorption in the kidney. Currently, a variety of molecules are found to be involved in the process of urine concentration by regulating the expression or activity of AQPs, such as antidiuretic hormone, renin-angiotensin-aldosterone system (RAAS), prostaglandin, and several nuclear receptors. As the main bile acid receptors, farnesoid X receptor (FXR) and membrane G protein-coupled bile acid receptor 1 (TGR5) play important roles in bile acid, glucose, lipid, and energy metabolism. In the kidney, FXR and TGR5 exhibit broad expression across all segments of renal tubules, and their activation holds significant therapeutic potential for numerous acute and chronic kidney diseases through alleviating renal lipid accumulation, inflammation, oxidative stress, and fibrosis. Emerging evidence has demonstrated that the genetic deletion of FXR or TGR5 exhibits increased basal urine output, suggesting that bile acid receptors play a critical role in urine concentration. Here, we briefly summarize the function of bile acid receptors in renal water reabsorption and urine concentration.

## Introduction

The kidney is a vital organ that receives 20%–25% of an adult human’s cardiac output and controls the metabolism of salt and water. About 180 L of filtrate are produced daily by an adult human kidney, but only 1–2 L of urine are expelled, with 99% of the primary urine being reabsorbed along various renal tubules.

Many factors are involved in the progress of urine concentration. Firstly, the hypertonic environment of the inner medullary is a necessary condition for urine concentration, which promotes the reabsorption of water. The hypertonic environment is mainly due to the accumulation of urea and sodium chloride in the inner medullary, where Na^+^/K^+^/2Cl^–^cotransporter (NKCC2) and urea transporters play an important role in this process. Secondly, aquaporins also play important roles in this process. Eight aquaporins, including AQP1-7 and AQP11, have been identified in the kidney (Noda et al., 2010). Among them, AQP1 is expressed on both the apical and basolateral membrane of the proximal tubule, as well as the thin descending limb of the loop of Henle and the vasa recta (Chou et al., 1999), responsible for the reabsorption of about 80% of the water in primary urine. AQP2 is highly expressed in the apical membrane and subapical vesicles of principal cells in the renal collecting duct, responsible for about 20% of water reabsorption (Kwon et al., 2013). Mice that do not have AQP1, AQP2, AQP3, or AQP4 genes exhibit a significant increase in urine production (Yang et al., 2001). Finally, The Epithelial sodium channel (ENaC) is important for sodium and water reabsorption, which is located in the apical membrane of the collecting ducts. ENaC facilitates Na⁺ reabsorption and then the movement of Na^+^ creates an osmotic gradient, allowing water to follow in the same direction.

Currently, it has been reported that a variety of molecules are involved in regulating the reabsorption of water in the kidney. Antidiuretic hormone (ADH) or arginine vasopressin (AVP) is a critical hormone synthesized in the hypothalamus that plays a key role in water homeostasis. In the renal collecting duct, AVP binds to the V2 receptor (V2R) and increases the phosphorylation of AQP2 by the cAMP-PKA pathway. Recently, increasing evidence has demonstrated that AVP played a critical role in facilitating urinary concentration via activating ENaC (Mironova et al., 2012; Mironova et al., 2015; Stockand et al., 2022; Wang et al., 2022). Moreover, AVP also rapidly increased water and urea transport in the terminal inner medullary collecting duct (IMCD) by increasing the expression and apical membrane trafficking of the urea transporter A1 (UT-A1) (Sands et al., 2011). The renin-angiotensin-aldosterone system (RAAS) additionally contributes to the regulation of water and sodium reabsorption in the kidney. Angiotensin II has different effects on different parts of the kidneys. In the proximal tubules, it heightens the activity of sodium/hydrogen exchanger and sodium-bicarbonate cotransporter through binding to the Angiotensin II receptor type 1 (AT1) receptor. In the distal tubule and collecting ducts, Angiotensin II further amplifies the activity of sodium-chloride cotransporter (NCC) and ENaC, which increases the reabsorption of water and sodium. Additionally, Angiotensin II increases aldosterone levels, which promotes sodium reabsorption by binding to the mineralocorticoid receptor (Harrison-Bernard, 2009; Zaika et al., 2013). Prostaglandin E2 (PGE2) is the main cyclooxygenase metabolite of arachidonic acid (AA). It is primarily synthesized in the medullary collecting tubule of the kidney (Bonvalet et al., 1987). EP1-4 are the 4 G protein-coupled receptors (GPCRs) of PGE2. Among these, EP1 and EP3 have high expression in the basolateral membrane of the collecting duct, respectively. When these receptors are activated, less sodium chloride and water are absorbed, which increases the excretion of sodium ions and urine (Ando and Asano, 1995; Guan et al., 1998; Breyer and Breyer, 2001; Nasrallah et al., 2018). In the kidney, EP2 expression is low, however, the activation of EP2 increased AQP2 membrane targeting (Olesen et al., 2016). The primary function of EP4, which is mostly expressed in glomeruli, is to control the release of renin (Breyer and Breyer, 2001). In the collecting ducts, disruption of EP4 impaired urinary concentration via decreasing AQP2 through the cAMP/PKA pathway (Gao et al., 2015). Several nuclear receptors have been implicated in regulating water homeostasis. Activation of peroxisome proliferator-activated receptor γ (PPARγ) increased the water and sodium reabsorption through the ENaC and AQP2 (Guan et al., 2005; Zhou et al., 2015). The activation of the glucocorticoid receptor (GR) enhanced the AQP2 gene expression induced by AVP in the collecting duct cell (Su et al., 2020). Liver X receptor β (LXRβ) knockout mice also exhibited polyuria due to decreased AVP and AQP1 (Gabbi et al., 2012), and increased ubiquitination of AQP2 protein (Su et al., 2017). The administration of estradiol decreased the expression of AQP2 by binding to estrogen receptor α, consequently leading to an increase in urine output in ovariectomized rats (Cheema et al., 2015).

Increasing evidence suggests that bile acid receptors play a crucial role in regulating renal water and sodium reabsorption. In this article, we will review the general functions of two bile acid receptors, nuclear receptor FXR, and membrane receptor TGR5, and finally focus on their roles in renal water and sodium homeostasis, offering novel strategies for addressing disorders related to water and salt metabolism, such as diabetes insipidus.

### Classification and general function of bile acid receptors

The liver is responsible for the synthesis of primary bile acids, specifically cholic acid (CA) and chenodeoxycholic acid (CDCA). These primary bile acids are subsequently conjugated and excreted into the intestine. Within the gut, they were metabolized by the gut microbiota, leading to the formation of secondary bile acids, namely, lithocholic acid (LCA) and deoxycholic acid (DCA) (Wang et al., 1999). In the intestine, roughly 95% of bile acids are reabsorbed into hepatocytes, with only a small fraction entering the bloodstream. Initially, these circulating bile acids go through glomerular filtration but are nearly completely reabsorbed in the proximal renal tubules, facilitated by the apical sodium-dependent bile acid transporter (ASBT) and the basolateral Organic Solute Transporter α/β (OSTα/β). Consequently, only about 5% of the filtered bile acids end up in the urine each day (Stiehl, 1974; Herman-Edelstein et al., 2018). Bile acids play crucial roles in various physiological and pathophysiological processes by binding and activating the nuclear receptor FXR and the membrane G protein-coupled receptor TGR5.

FXR is a member of the nuclear receptor (NR) superfamily that controls the transcription of specific target genes. It exhibits prominent expression in organs such as the liver, kidney, and small intestine. As the endogenous ligand of FXR, bile acids activate FXR in the following order: CDCA > DCA > LCA > CA (Wang et al., 1999). Upon activation, FXR assumes critical roles in the regulation of various metabolic pathways, including bile acid, glucose, and lipid metabolism (Guo et al., 2023). Notably, it acts to inhibit the production and accumulation of bile acids in the liver and intestines (Liu et al., 2003; Boyer et al., 2006; Landrier et al., 2006). Additionally, the activation of FXR increases glycogen synthesis and reduces glycolysis (Zhang et al., 2006; Caron et al., 2013; Dong et al., 2019). Furthermore, it reduced the accumulation of lipids in the kidney in insulin-resistance animal models (Nakahara et al., 2002; Zhang et al., 2006; Lai et al., 2022). Recently, several studies reported that overexpression of FXR in the kidney substantially alleviated hypertension and elevated renal nitric oxide (NO) levels, which was achieved by stimulating the expression of endothelial nitric oxide synthase (eNOS) in a mouse model of hypertension induced by an 8-week regimen of 20% fructose in drinking water combined with a 4% sodium chloride diet (referred to as HFS) (Ghebremariam et al., 2013; Li et al., 2015). In the kidney, activation of FXR attenuated acute kidney injury caused by cisplatin and renal ischemia–reperfusion (I/R) through regulating apoptosis, ferroptosis, and autophagy (Bae et al., 2014; Gai et al., 2017; Luan et al., 2021; Zhang et al., 2022). Additionally, FXR agonists also improved renal inflammation, fibrosis, lipid accumulation, and glucose metabolism disorders, which prevented the progression of chronic kidney disease (Evans et al., 2009; Wang et al., 2010; Zhou et al., 2016; Marquardt et al., 2017).

TGR5 is a membrane receptor of bile acids, which can be bound and activated by various endogenous bile acids, especially LCA. TGR5 is expressed in various tissues, such as the kidney, liver, digestive tract, and central nervous system (Poole et al., 2010). Many studies have demonstrated that it plays a significant role in multiple physiological processes, as well as the pathogenesis of various metabolic diseases (Reich et al., 2021; Tian et al., 2022; Chen et al., 2023). Activation of TGR5 results in coupling with a stimulatory G-alpha-protein (Gαs) which, in turn, activates the cAMP-PKA signaling pathway. This cascade of events results in the phosphorylation of the cAMP response element binding protein (CREB) and its subsequent nuclear import, ultimately leading to the activation of the target genes. When activated, TGR5 promotes GLP-1 secretion from enteroendocrine L cells (Li et al., 2017), mitochondrial thermogenesis in adipocytes (Velazquez-Villegas et al., 2018), and protected against lipopolysaccharide (LPS)-induced liver inflammation by decreasing inflammatory cytokine secretion (Wang et al., 2011). In the kidney, TGR5 activation has been found to alleviate renal I/R injury by reducing inflammation and macrophage migration (Zhang et al., 2019). Furthermore, it has also been observed that the activation of TGR5 can prevent renal inflammation and fibrosis by inhibiting the NF-κB pathway in diabetic mice induced by streptozotocin (STZ) (Xiao et al., 2020). Moreover, a selective TGR5 agonist INT-777 has been shown to ameliorate proteinuria and podocyte injury in diabetic db/db mice (Wang et al., 2016). In addition, TGR5 activation also decreased the high glucose-induced fibrosis in glomerular mesangial cells (GMCs) (Yang et al., 2016). Recently studies have also revealed that the deubiquitination of TGR5 at K306 residue also restored TGR5 expression and protected db/db mice from diabetic nephropathy (Lin et al., 2023).

Currently, there are some studies on dual FXR and TGR5 agonists in the kidney. Diabetic mice treated with the dual FXR/TGR5 agonist INT-767 showed an improvement in proteinuria and prevention of podocyte injury, mesangial expansion, and tubulointerstitial fibrosis (Wang et al., 2016). The same dual agonist reduced the proteinuria and fibronectin accumulation in aging mice (Wang et al., 2017). These findings indicate a potential role of dual FXR/TGR5 agonists in the regulation of many kidney diseases.

### Bile acid receptors and kidney water homeostasis

As mentioned above, about 95% of the bile acids synthesized from the liver are recycled through the enterohepatic circulation. In the kidney, approximately 100 μmol bile acids are filtered in the glomeruli per day, and almost all of them are reabsorbed in the proximal tubule. Only 1–2 μmol/day is excreted in the urine (Dawson et al., 2009). The renal tubules, especially the collecting duct, express high levels of bile acid receptors FXR and TGR5, where water is reabsorption through aquaporins to complete the final step of urine concentration, suggesting that FXR and TGR5 may play an important role in the regulation of water homeostasis. Whether the activation of the bile acid receptor in collecting ducts plays a significant role in water homeostasis is worth studying.

### Farnesoid X receptor and renal water reabsorption

In the kidney, FXR exhibits high expression levels in various renal tubules, especially the collecting duct. However, the current study of FXR in water reabsorption only reveals its role in collecting ducts. FXR plays a pivotal role in the regulation of urine volume. FXR knockout mice displayed diminished urine concentrating ability in comparison to WT mice, and its activation by binding with CDCA or a synthetic agonist GW4064 increased urinary concentrating capacity, mainly by increasing renal AQP2 expression (Zhang et al., 2014). Moreover, MCDs are exposed to a massive hypertonic environment, which is critical in regulating urine concentration. FXR can prevent hypertonic-induced apoptosis of MCDs by activating tonicity response enhancer-binding protein (TonEBP), a critical transcription factor responsible for facilitating the cellular accumulation of organic osmolytes to resist the hyperosmotic stress through increasing the expression of the target genes, including aldose reductase (AR) and heat shock protein 70 (HSP70) (Xu et al., 2018). Recently, studies revealed that crystallin zeta (CRYZ), a direct target gene of FXR, increased the NKCC2 expression to help maintain medullary hyperosmotic gradient. Additionally, overexpressing CRYZ reduced the cell death caused by hypertonicity by elevating the expression of B-cell lymphoma 2 (BCL2). These data demonstrated that FXR plays a critical role in the regulation of urine volume by increasing the expression of AQP2 and promoting the survival of MCDs in a dehydrated state. The above results proved the physiological function of FXR in water reabsorption, but its role in the pathophysiological state such as diabetes insipidus is still unclear. Moreover, recent evidence also showed that enhanced urinary excretion of bile acids in some conditions such as cholestasis may cause the injury of tubular epithelial cells, and FXR agonist obeticholic acid (OCA) ameliorated the renal tubular damage in bile duct ligation (BDL) induced hepatorenal syndrome (HRS) (Tsai et al., 2020). Low urine volume also existed in HRS mainly caused by a reduction in renal blood flow, it is not known the effect of increased bile acids in renal tubules on the expression of AQPs and water reabsorption.

### TGR5 and renal water reabsorption

In normal kidney tissue, TGR5 exhibited high expression in collecting ducts, distal convoluted tubules, and the thin loop of Henle, with minimal or sporadic weak staining in the proximal tubules (Zhao et al., 2018). Different from the study of FXR in water reabsorption focusing on physiological levels, the study of TGR5 in AQP2 regulation is mainly carried out in kidney diseases. Lithium is a frequently prescribed medication for managing bipolar disorder, which may cause multiple endocrinopathies including nephrogenic diabetes insipidus (NDI). In a mouse model of lithium-induced nephrogenic diabetes insipidus, the activation of TGR5 by INT-777 or INT-767 elevated the expression of AQP2 through the cAMP/PKA signaling pathway (Li et al., 2018). Acute kidney injury is a common clinical disease, accompanied by changes in urine output. With the progression of the disease, oliguria, anuria, and polyuria can occur. In the I/R-induced AKI rat model, urinary output was significantly decreased, accompanied by the loss of renal AQP1, AQP2, and AQP3 in the cortex and outer medulla (Hussein et al., 2012; Asvapromtada et al., 2018; Liu et al., 2021). While the activation of TGR5 by LCA or INT-777 effectively prevented the downregulation of renal AQP2 in I/R-induced kidney injury through activating HIF-1α signaling (Han et al., 2021). These findings support the potential role of TGR5 in the regulation of renal water reabsorption.

## Perspectives

**FIGURE 1:**
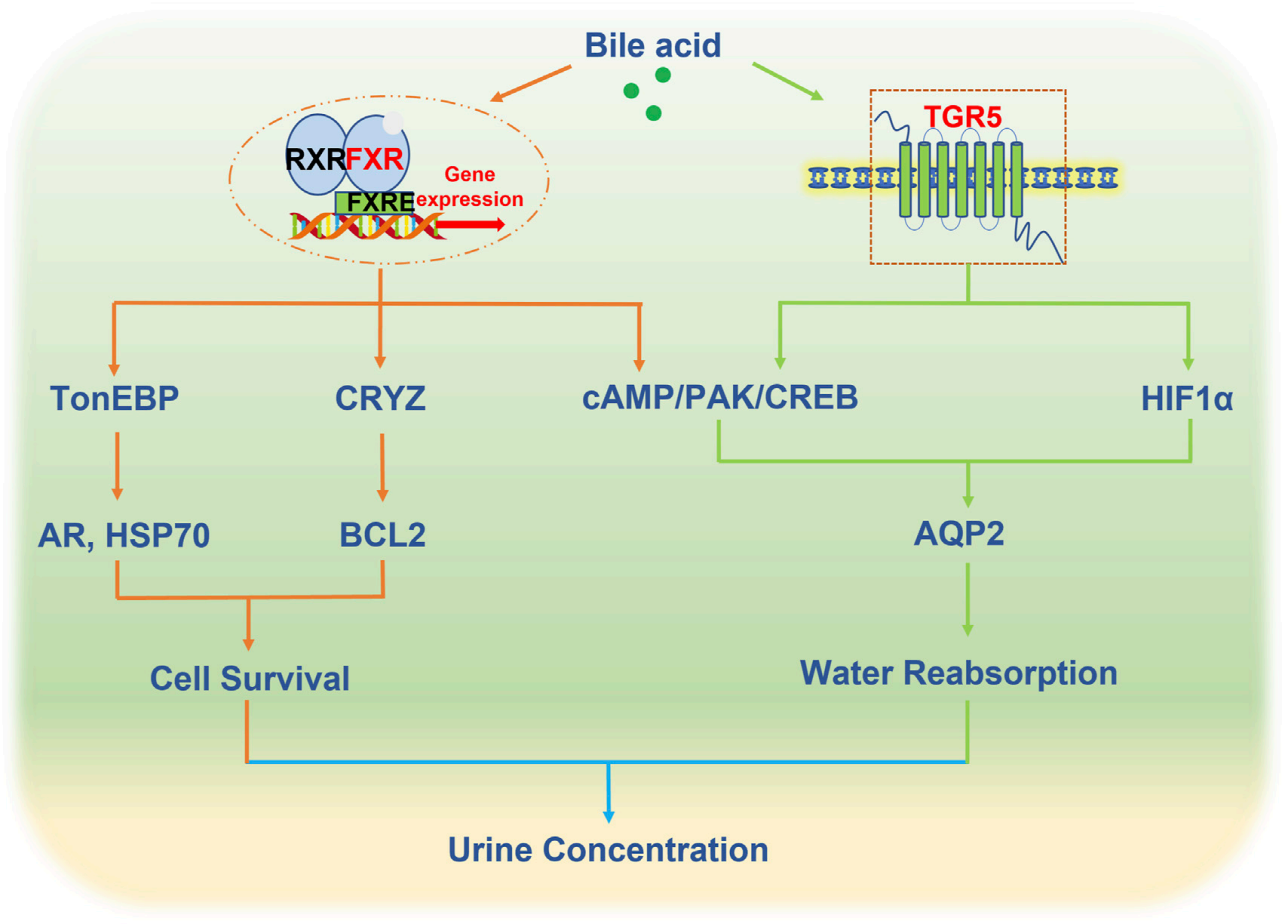
Bile acid receptors in the regulation of urine concentration.

FXR and TGR5, as bile acid receptors, play an important role in renal water homeostasis through regulating the expression and trafficking of AQP2, which provides a novel therapeutic for the treatment of water and salt metabolism disorders such as diabetes insipidus (Figure 1). However, the mechanism of bile acids in the regulation of water homeostasis is largely unknown. Firstly, there are 8 types of aquaporins in the kidney, in addition to affecting AQP2, whether the activation of bile acid receptors affects other aquaporins is worth exploring. Secondly, AVP synthesized in the hypothalamus regulates AQP2 expression. Recently studies revealed that FXR and TGR5 were expressed in the hypothalamus (Castellanos-Jankiewicz et al., 2021; Deckmyn et al., 2021), and circulating bile acids can also reach the hypothalamus (Xu, 2018). Whether bile acid receptors affect AVP secretion remains to be investigated. Thirdly, in addition to aquaporins, other ion channels such as ENac can also indirectly affect water reabsorption. At present, research has not delved into the direct regulation of ENaC by either FXR or TGR5 in the kidney, but several studies have reported that ENaC is regulated by bile acids in overexpressed human ENaC *Xenopus laevis* oocytes (Ilyaskin et al., 2016; Wang et al., 2019).
